# Novel Roles for Geranylgeranyl Transferase-III (GGTase-III) in Insulin Secretion

**DOI:** 10.33594/000000783

**Published:** 2025-06-30

**Authors:** Noah F. Gleason, Mirabela Hali, Anjaneyulu Kowluru

**Affiliations:** a Biomedical Research Service, John D. Dingell VA Medical Center and Department of Pharmaceutical Sciences, Wayne State University, Detroit, MI 48201; b Division of Metabolism, Endocrinology & Diabetes, University of Michigan Medical School, Ann Arbor, MI 48109

**Keywords:** Protein prenylation, GGTase-III, Islet β-cell, Insulin secretion, G proteins, Diabetes

## Abstract

**Background/Aims:**

Post-translational prenylation of G proteins is implicated in physiological insulin secretion. It has been reported recently that GGTase-III participates in the functional regulation of Ykt6, a synaptobrevin homolog, *via* geranylgeranylation. However, potential localization and putative regulatory roles of GGTase-III in insulin secretion remains unknown. The current study is aimed at determining the expression and contributory roles of GGTase-III in glucose- and KCl-induced insulin secretion from pancreatic β-cells.

**Methods:**

Mouse islets were isolated by the collagenase digestion method. Human islets were from Prodo Laboratories. INS-1 832/13 cells were transfected with either control (scrambled) or siRNA-PTAR1 (the α-subunit of GGTase-III) using lipofectamine RNAiMax. Insulin released into the medium was quantified using a commercially available Insulin ELISA kit. Expression of GGTase-III subunits and ykt6 was determined by Western blotting and quantified by densitometry.

**Results:**

Western blotting revealed that both subunits of GGTase-III (PTAR1 and RabGGTB) are expressed in human islets, mouse islets and INS-1 832/13 cells. Transfection of INS-1 832/13 cells with siRNA-PTAR1 resulted in significant reduction (~50%) in the expression of PTAR1. siRNA-mediated knockdown of PTAR1 significantly attenuated (~60%) glucose-stimulated insulin secretion (GSIS) in INS-1 832/13 cells. Furthermore, insulin secretion elicited *via* KCl-induced membrane depolarization was markedly reduced (~69%) in INS-1 832/13 cells following PTAR1 depletion. Lastly, immunoblotting data suggested expression of Ykt6, a known substrate for GGTase-III, in human islets, rodent islets, and INS-1 832/13 cells.

**Conclusion:**

A GGTase-III-dependent signaling step is necessary for glucose- and KCl-induced insulin secretion.

## Introduction

**Fig. 1.**
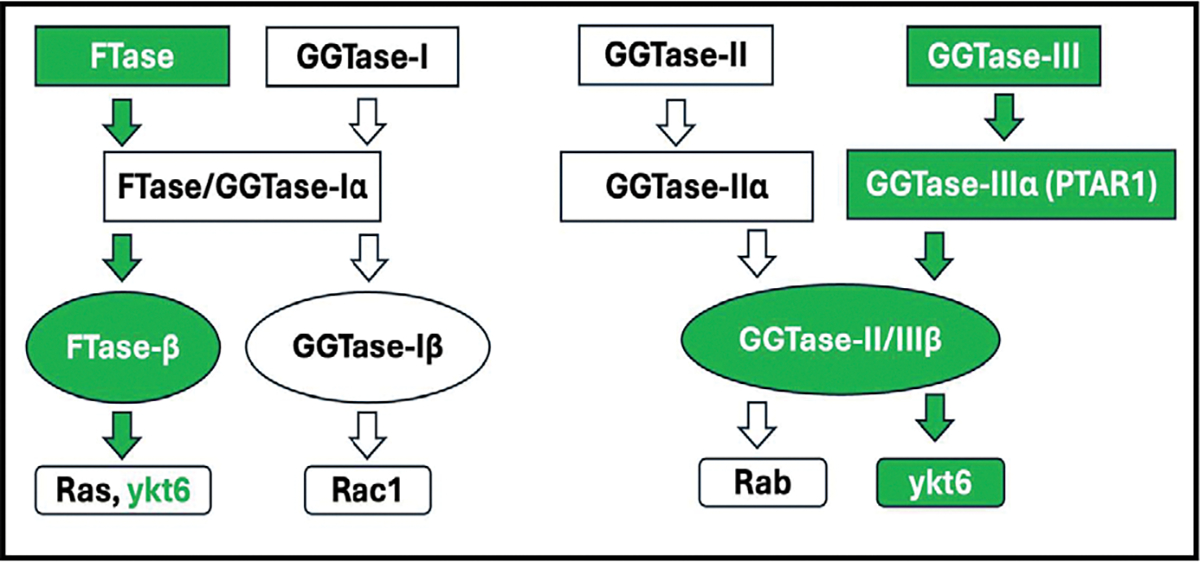
Schematic depiction of subunit composition of prenyltransferases and their substrates: At least four types of prenyltransferases, namely FTase-I, GGTase-I, GGTase-II and GGTase-III have been identified in mammalian cells. They exist as heterodimers, comprising of α- and β-subunits. As highlighted in the Fig., FTase and GGTase-I share a common α-subunit, but distinct β-subunits. GGTase-II and GGTase-III (focus of this studies; highlighted in green) share common β-subunit, but distinct α-subunits. PTAR1 represents the α-subunit of GGTase-III. FTase mediates farnesylation of a variety of proteins, including Ras, nuclear lamins and ykt6 (focus of these studies; highlighted in green). GGTase-I promotes geranylgeranylation of a smgs belonging to Rho subfamily, including Rac1, Cdc42 and RhoA. GGTase-II mediates geranylgeranylation of smgs belonging to Rab subfamily. GGTase-III is involved in geranylgeranylation of ykt6 and FBXL2.

Published evidence from multiple laboratories implicates novel regulatory roles for small molecular weight G proteins (smgs; e.g., Arf6, Cdc42, Rac1, Rab3A and Rab27A) in pancreatic islet β-cell function including glucose-stimulated insulin secretion (GSIS) [1–4]. Data accrued from extant investigations have demonstrated critical roles for post-translational modifications (e.g., prenylation) of smgs in GSIS [5–7]. Briefly, post-translational prenylation is mediated by at least three prenyltransferases, namely the farnesyltransferase (FTase) and the geranylgeranyl transferases-I and II (GGTases-I and -II; Fig. 1). The FTase and GGTases (GGTase-I and -II) facilitate incorporation of a 15-carbon farnesyl or a 20-carbon geranylgeranyl groups into the C-terminal cysteines (CAAX motif) of smgs, respectively. The FTase facilitates farnesylation of smgs, including Ras, whereas GGTase-I and -II promote geranylgeranylation of Rho (e.g., Cdc42, Rac1, Rho A) and Rab (Rab3A and Rab27A) subfamilies of smgs, respectively. The FTase and GGTase-I and -II are heterodimeric in composition comprising of α- and β-subunits [2, 8]. Interestingly, the FTase and GGTase-I share a common α-subunit but are composed of distinct β-subunits (Fig. 1). The GGTase-II, which geranylgeranylates the Rab subfamily of smgs, is also heterodimeric comprised α- and β-subunits that are distinct from the α and β subunits of FTase and GGTase-I. Pharmacological and molecular biological evidence suggests critical roles for FTase, GGTase-I and -II in GSIS [2, 6, 9, 10].

More recently, GGTase-III, a fourth prenyltransferase, has been shown to mediate the geranylgeranylation of YKT6 v-SNARE homolog protein (ykt6) and F-Box and Leucine Rich Repeat Protein 2 (FBXL2) [11, 12]. As in the case of FTase, GGTase-I and II, the GGTase-III is also heterodimeric comprising of the α- (protein prenyltransferase α subunit repeat containing 1; PTAR1) and β- (Rab geranylgeranyl transferase β; RabGGTB)-subunits. Furthermore, the α-subunit of GGTase-III is distinct from the α-subunits of FTase/GGTase-I and GGTase-II. Interestingly, however, the β-subunit of GGTase-III is shared by the GGTase-II (Fig. 1).

Despite the emerging evidence in other cell types on its role in mediating the prenylation of ykt6 and FBXL2 and downstream signaling events [13–15], the expression and putative regulatory roles of GGTase-III in pancreatic islet β-cell function, including insulin secretion has not been assessed thus far. Therefore, the current study is undertaken to determine the expression of α- and β- subunits of GGTase-III in INS-1 832/13 cells, rodent and human islets, and then to assess putative roles of GGTase-III in promoting insulin secretion elicited by glucose and a membrane depolarizing concentration of KCl. Data accrued in these investigations demonstrate for the first time that GGTase-III plays novel roles in insulin secretion elicited by glucose and KCl.

## Materials and Methods

### Materials

Antibody directed against PTAR1 was from MyBiosource (San Diego, CA, USA). Anti-RabGGTTB was from Proteintech (Rosemont, IL, USA) and anti-ykt6 serum was from Santa Cruz Biotechnology (Dallas, TX, USA). Anti-GAPDH and HRP-conjugated rabbit secondary antibody were from Cell Signaling Technologies (Danvers, MA, USA). siRNA for PTAR1 and scrambled (control) siRNA were acquired from Dharmacon (Lafayette, CO, USA). Lipofectamine RNAiMax and transfection reagent Opti-MEM reduced serum media were purchased from ThermoFischer Scientific (Carlsbad, CA, USA). Insulin ELISA kit was from ALPCO (Salem, NH, USA).

### Mouse islets and human islets, and culture of INS-1 832/13 cells

Mouse islets were isolated by the collagenase digestion method [6, 16]. Human islets were from Prodo Laboratories (Aliso Viejo, CA). INS-1 832/13 cells were from Sigma Aldrich (St. Louis, MO, USA). INS-1 832/13 cells were cultured in RPMI-1640 media supplemented with 10% fetal bovine serum (FBS), 11.1 mM D-glucose, 100 IU/mL penicillin and streptomycin, 1 mM sodium pyruvate, 50 μM 2-mercaptoethanol, and 10 mM HEPES (pH adjusted to 7.4). Cells were sub-cloned twice weekly. Cells between passages 5–11 were utilized in these investigations, including determination of glucose- and KCl-induced insulin secretion. Prior to stimulation with glucose or KCl, cells were starved overnight in low serum/low glucose media (LS/LG media; 2.5 mM glucose and 2.5% FBS) [17].

### siRNA-mediated knockdown of PTAR1

Endogenous expression of PTAR1 in INS-1 832/13 cells was suppressed by siRNA transfection following manufacturer’s protocol. Scrambled siRNA duplexes were used as control. Transfected cells were maintained in growth medium mix containing no antibiotic for 72 hours prior to exposure to any treatment conditions. Degree of PTAR1 depletion was confirmed by Western blotting and quantified by densitometry.

### Western blotting

After treatment, cells were collected and lysed in RIPA lysis buffer (with protease and phosphatase inhibitors). Lysate proteins were separated by SDS-PAGE and then transferred to nitrocellulose membranes. Membranes were blocked in 3% BSA for 1 hour at room temperature with constant shaking. Membranes were then incubated with specific primary antibody prepared in PBS-T containing 1.5% BSA. (1:1000 dilution of antisera for PTAR1, RGGTB, and ykt6; 1:5000 for GAPDH) overnight at 4° C with constant shaking. The next day, membranes were washed with PBS-T (3 times for 5 minutes each) and then incubated with rabbit conjugated secondary antibody (1:2000) for 1 hour at room temperature. Membranes were then washed again with PBS-T (3 times; 5 minutes each). Proteins were visualized with Pierce ECL Substrate (ThermoFisher Scientific) and relative protein expression quantified by densitometry.

### Insulin secretion assay

Cells transfected with either scrambled control-si or PTAR1-si were incubated overnight in LS/LG media. Prior to the treatment, the cells were then incubated in Krebs Ringer Bicarbonate buffer (KRB, pH 7.4) for 1 hour at 37° C. Cells were then incubated in KRB supplemented with either low glucose (LG, 2.5 mM) or high glucose (HG, 20 mM) for 45 minutes. For KCl stimulation studies, cells were pre-incubated in KRB for 1 hour and then treated with either LG (2.5 mM) or KCl (60 mM) for 1 hour. At mentioned time points, insulin released into the media was quantified using an insulin ELISA kit per the manufacturer’s protocol. The data are expressed as fold change relative to LG-Con-si as in [16–18].

### Statistical analysis of experimental data

Data were analyzed using GraphPad Prism 9.5 (GraphPad Software; San Diego, CA, USA). Data are shown as mean ± standard error of mean (SEM) from multiple independent studies. A two-tailed Student t-test was used to compare two groups while a one-way analysis of variance (ANOVA) and Tukey’s multiple comparison was utilized when comparing more than two groups. A p value less than 0.05 was considered significant.

## Results

**Fig. 2.**
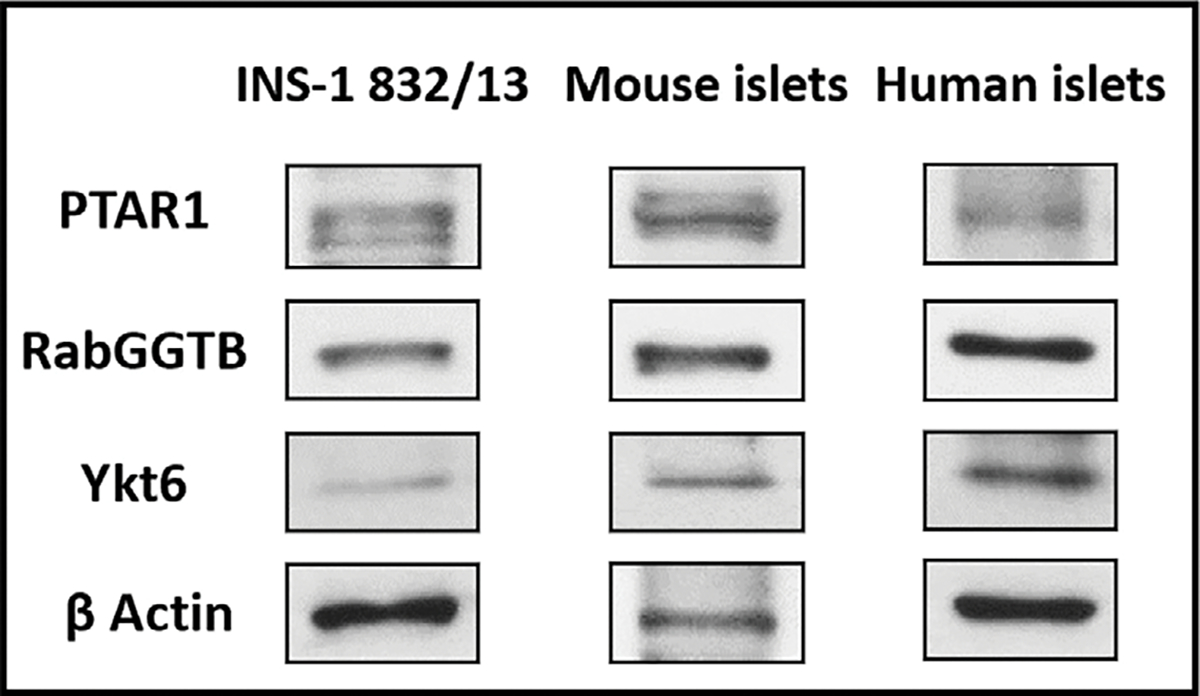
Immunological evidence for the expression of α- (PTAR1) and β-(RabGGTB) subunits of GGTase III and ykt6 in INS-1 832/13 cells, mouse islets and human islets. Lysates derived from INS-1 832/13 cells, mouse islets and human islets were employed for immunodetection of the α-(PTAR1) and β-(RabGGTB) and ykt6 by Western blotting. β-actin was used as loading control.

At the outset, we determined the expression of the α-(PTAR1) and β-(RabGGTB) subunits of GGTase-III in INS-1 832/13 cells, rat islets and human islets. Data depicted in Fig. 2 suggest that both subunits of GGTase III are expressed in all the three cell types studied. In addition, data in Fig. 2 provided the first evidence for the expression of ykt6, a synaptobrevin analog and a known substrate for GGTase III, in all the three cell types.

**Fig. 3.**
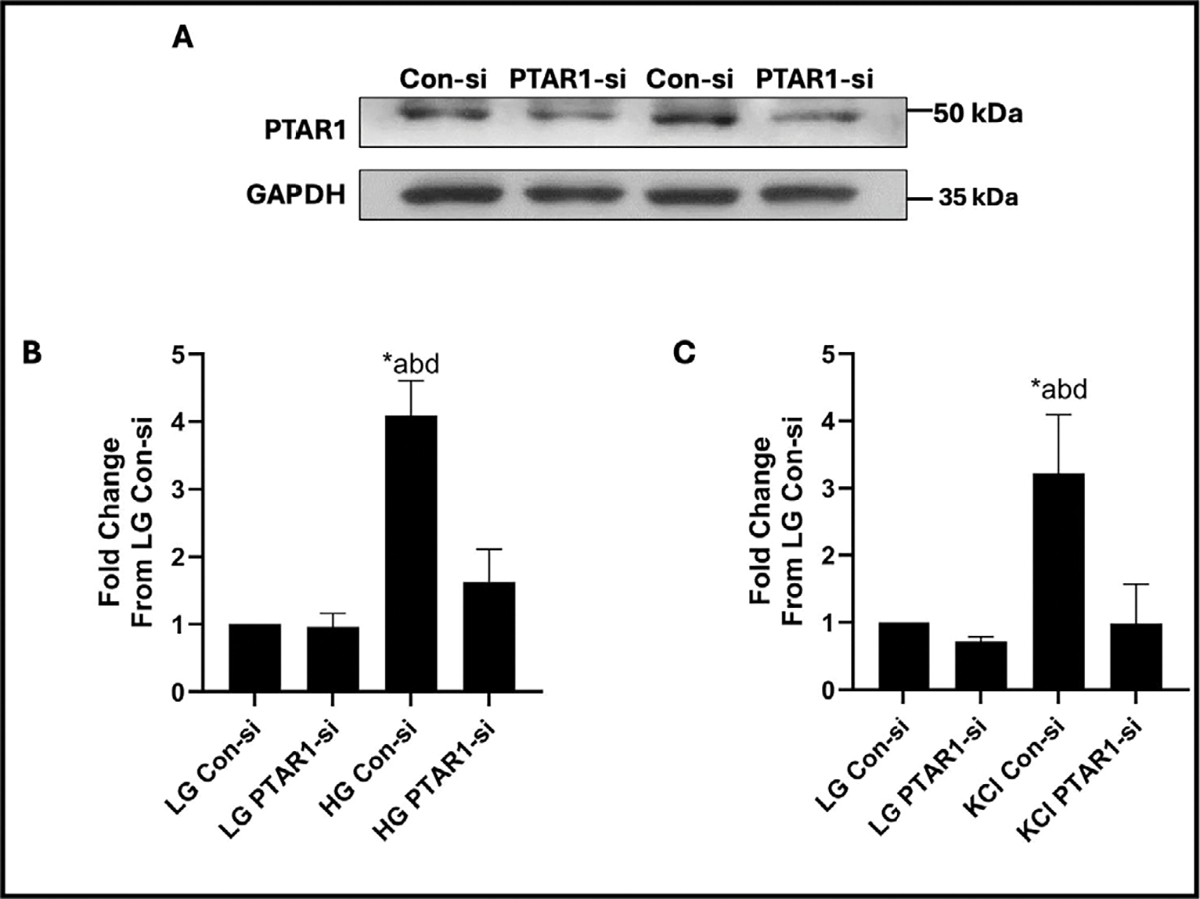
Depletion of endogenous expression of PTAR1 attenuates glucose- and KCl-induced insulin secretion from INS-1 832/13 cells. Panel A: siRNA-mediated knockdown of PTAR1 in INS-1 832/13 cells: Cells were transfected with either scrambled siRNA (Con-si) and siRNA-PTAR1 (100 nM each) using lipofectamine RNAiMax transfection reagent. The cells were incubated for 72 hours in media containing no antibiotic and Opti-MEM mix to achieve optimal protein depletion. Representative blot showing transfections from duplicate studies is shown here. Panel B: siRNA-mediated knockdown of PTAR1 markedly attenuates GSIS in INS-1 832/13 cells: Con-si or PTAR1-si transfected INS-1 832/13 cells were incubated under low (LG; 2.5 mM) or high (HG; 20 mM) glucose for 45 minutes. Insulin released into the medium was quantified using a commercially available insulin ELISA detection kit. Data are presented as mean ± SEM from four independent studies, each sample having 2 replicates. Lane a: cells transfected with Con-si and incubated with LG; lane b: cells transfected with siRNA-PTAR1 and incubated with LG; lane c: cells transfected with Con-si and incubated with HG; and lane d: cells transfected with siRNA-PTAR1 and incubated with HG. *p<0.05. Panel C: siRNA-mediated depletion of PTAR1 markedly inhibits KCl-induced insulin secretion in INS-1 832/13 cells: Con-si or PTAR1-si transfected INS-1 832/13 cells were exposed to low glucose (LG; 2.5 mM) or KCl (60 mM) for 60 minutes. Insulin released into the medium was quantified using a commercially available insulin ELISA detection kit. Data are presented as mean ± SEM from four independent studies, each sample having 2 replicates. Lane a: cells transfected with Con-si and incubated with LG; lane b: cells transfected with siRNA-PTAR1 and incubated with LG; lane c: cells transfected with Con-si and exposed to KCl; and lane d: cells transfected with siRNA-PTAR1 and exposed to KCl. *p<0.05.

The next set of studies were aimed at determining putative roles of GGTase III in insulin secretion elicited by an insulinotropic concentration of glucose. To accomplish this, we first optimized conditions for deletion of PTAR1 expression in INS-1 832/13 cells using siRNA-PTAR1. Data in Fig. 3A demonstrate 57% reduction in the expression of PTAR1 following transfection of siRNA-PTAR1. We used these conditions to determine potential impact of siRNA-mediated knockdown of PTAR1 on GSIS. Data shown in Fig. 3B indicate four-fold stimulation of insulin secretion in INS-1 832/13 cells exposed to stimulatory glucose (bar 1 vs. bar 3). siRNA-mediated depletion of PTAR1 had no significant effect of basal insulin secretion in these cells (bar 1 vs bar 2). A significant inhibition of GSIS (~ 60%) was noted in these cells following knockdown of PTAR1 (bar 3 vs. bar 4). Together, these findings provide the first evidence for critical regulatory roles for GGTase III in physiological insulin secretion.

We next determined roles of GGTase III in insulin secretion from INS-1 832/13 cells elicited by a membrane-depolarizing concentration of KCl. Data depicted in Fig. 3C demonstrate robust secretion of insulin from these cells following exposure to KCl (bars 1 vs. 3). In a manner akin to glucose-induced insulin secretion (Fig. 3B), KCl-induced secretion was also attenuated significantly in INS-1 832/13 cells following knockdown of PTAR1 expression. Together, based on the data shown in Fig. 3, we conclude that glucose-and KCl-induced insulin secretion might underlie signaling steps that require GGTase III-mediated prenylation of specific proteins.

## Discussion

A growing body of evidence suggests that protein prenylation is essential for intracellular events including cytoskeletal reorganization, and membrane trafficking of signaling proteins [19]. Herein, we provided the first immunological evidence for the expression of α- and the β-subunits of GGTase-III in INS-1 832/13 cells, rodent and human islets. Our findings also implicate critical roles for this novel prenyltransferase in glucose- and KCl-induced insulin secretion.

What are potential mechanisms that might contribute to GGTase-III mediated control of insulin secretion? We propose that they might involve prenylation of key signaling proteins, such as ykt6, which is expressed in clonal β-cells, mouse and human islets (Fig. 2). Ykt6, a SNARE protein, has been implicated in a variety of secretory, endocytotic and autophagic pathways. Published evidence implicates key roles for vesicle associated proteins (e.g., synaptobrevins) and membrane recognition and docking proteins in the cascade of events leading to glucose-induced cytoskeletal remodeling and vesicular transport leading to exocytotic secretion of insulin [20, 21]. Although it remains to be validated experimentally, GGTase-III mediated prenylation of ykt6 could contribute to the signaling steps involved in glucose- and KCl-induced insulin secretion (see below).

Available evidence suggests that ykt6 undergoes diprenylation at its conserved C-terminal cysteines. It has been shown that the C-terminal CCAIM motif of Ykt6 is farnesylated at the C195 residue by the FTase, prior to its geranylgeranylation at the C194 residue by the GGTase-III [13, 22, 23]. Along these lines, Pylypenko and coworkers [24] have suggested that FTase-mediated farnesylation of Ykt6 promotes its stability and helical folding. Compelling evidence indicates that GGTase-III mediated geranylgeranylation of Ykt6 at C194 is critical for optimal cellular functions regulated by ykt6. For example, using GGTase-III-deficient cells Shirakawa and coworkers have reported that ykt6 remained only in the farnesylated form leading to severe abnormalities in the Golgi-SNARE complex assembly. This, in turn, resulted in disorganized Golgi and associated delays in intra-Golgi protein trafficking. Based on these observations, it was concluded that double prenylation (farnesylation and geranylgeranylation) of Ykt6 is critical for the structural and functional organization of the Golgi apparatus [12]. Sakata and coworkers have demonstrated abnormalities in sorting of lysosomal hydrolases (cathepsin D and β-hexosaminidase) at the trans-Golgi network leading to their secretion into the extracellular space [22]. Together, these observations affirm critical roles for FTase, and GGTase-III facilitated diprenylation of ykt6 in optimal cell function. In addition to diprenylation, studies have shown that ykt6 undergoes phosphorylation of S174 within the SNARE domain to facilitate the conversion of Ykt6 from a closed to an open conformation thereby promoting its membrane association [13]. Lastly, ykt6 has also been shown to undergo palmitoylation and depalmitoylation, which appear to regulate their cycling between cytosolic and membrane compartments [25, 26].

Lastly, previous studies have reported contributory roles for ykt6 in the onset of diseases including cancer and Parkinson’s disease [27–29]. In the context of the islet β-cell, significant defects in the exocytotic proteins involved in insulin secretion (e.g., SNARE proteins) have been reported in diabetes [30, 31]. Additional investigations are needed to determine potential impact of diabetogenic conditions on the function of GGTase-III, and associated prenylation of ykt6, to further assess the roles of this signaling pathway in the onset of β-cell defects induced by metabolic stress. Indeed, potential impact of metabolic stress on protein prenylation in the onset of beta cell dysfunction has been documented [2, 32, 33]. These aspects of GGTase-III/ykt6 signaling module in islet β-cell function and dysregulation are being examined currently in our laboratory.

**Fig. 4.**
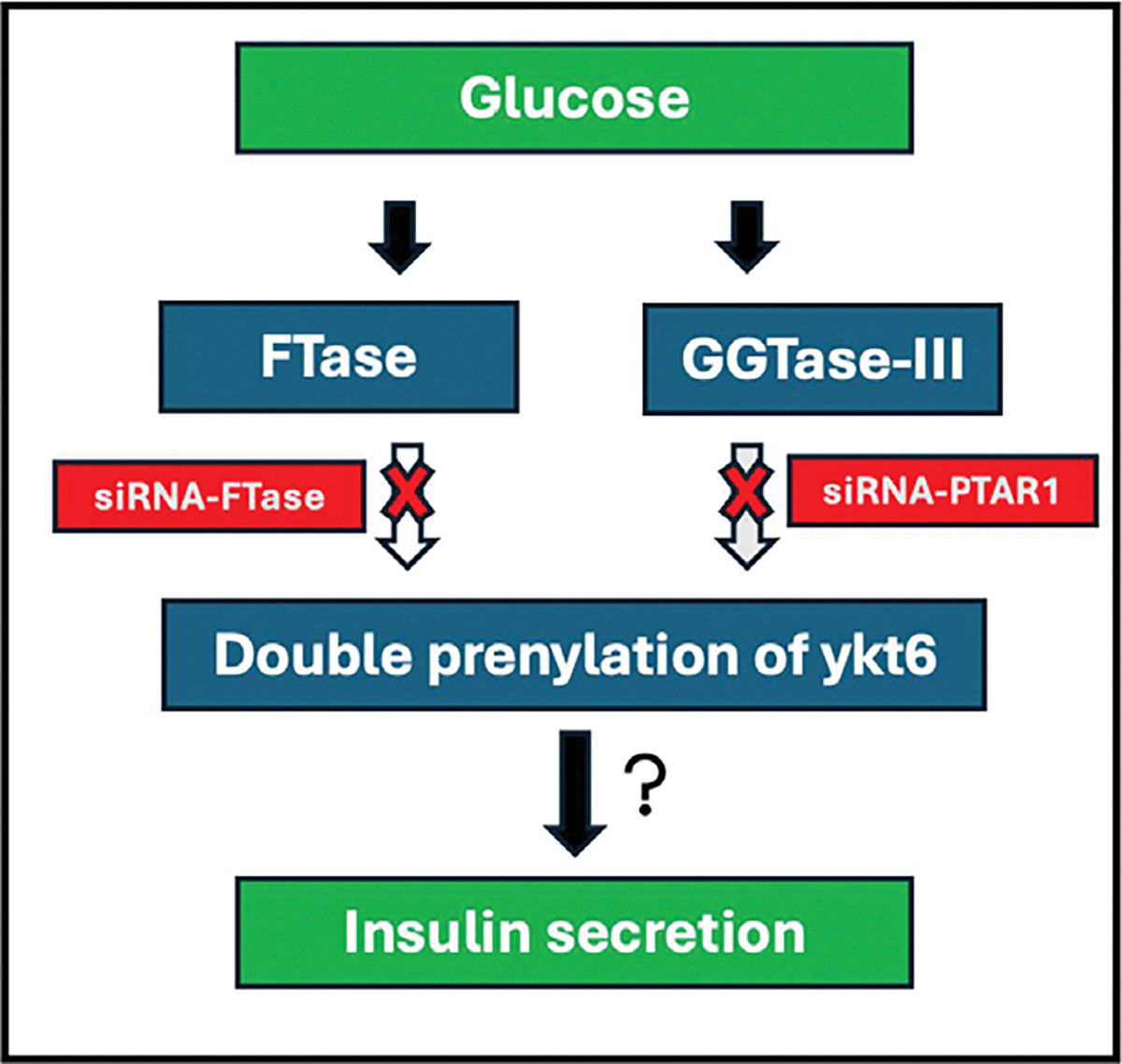
Schematic representation of potential mechanisms underlying FTase-GTTase-III mediated double prenylation of ykt6 leading to glucose-stimulated insulin secretion. Published evidence demonstrated an increase in the catalytic activities of prenyltransferases (FTase and GGTase-I) in INS-1 832/13 cells and rat islets under conditions of glucose-stimulated insulin secretion. Pharmacological and molecular biological evidence also implicates protein farnesylation is requisite for GSIS to occur. Data accrued from the current investigations suggest that siRNA-mediated knockdown of PTAR1 culminates in loss of GSIS. Based on these observations we propose that GSIS might involve activation of FTase and GGTase-III leading to double prenylation of ykt at C194 (geranylgeranylation) and C195 (farnesylation) thus favoring translocation and docking of insulin containing secretory granules at the plasma membrane for exocytotic secretion of insulin. Note that this remains to be validated experimentally (see text for additional details).

In conclusion, data from these studies provide first experimental evidence for the expression of α- and β-subunits of GGTase-III in human islets, mouse islets and clonal INS-1 832/13 β-cells. In addition, we presented evidence implicating GGTase-III in glucose- and KCl-induced insulin secretion. Putative regulatory roles of GGTase-III mediated prenylation of Ykt6 in insulin secretion module remain to be investigated in the future. Based on the available evidence we propose a working model for potential mechanistic connection between FTase-GTTase-III mediated double prenylation of ykt6 leading to glucose-stimulated insulin secretion (Fig. 4). Earlier studies by Goalstone and coworkers have reported a significant increase in the catalytic activities of prenyltransferases (FTase and GGTase-I) in INS-1 832/13 cells and rat islets under conditions of glucose-stimulated insulin secretion [34]. Pharmacological and molecular biological evidence also implicates protein farnesylation is requisite for GSIS to occur [2, 6]. Data accrued from the current investigations suggest that siRNA-mediated knockdown of PTAR1 results in inhibition of GSIS. Based on these observations we propose that GSIS might involve activation of FTase and GGTase-III leading to double prenylation of ykt at C194 (geranylgeranylation) and C195 (farnesylation) thus favoring exocytotic secretion of insulin. This needs to be validated experimentally.

## Abbreviations

FBXL2: F-Box (And Leucine Rich Repeat Protein 2)
FTase: farnesyl (transferase)
GGTase-I: geranylgeranyl (transferase-I)
GGTase-II: geranylgeranyl (transferase-II)
GGTase-III: geranylgeranyl (transferase-III)
GSIS: glucose-stimulated (insulin secretion)
PTAR1: Protein (Prenyltransferase Alpha Subunit Repeat Containing 1)
RabGGTB: β-subunit (of geranylgeranyl transferase-II)
smgs: small (molecular weight g proteins)
SNARE: Soluble (*N*-ethylmaleimide-sensitive factor attachment protein receptors)
Ykt6: YKT6 (v-SNARE homolog)
